# Impaired MC3T3-E1 osteoblast differentiation triggered by oncogenic HRAS is rescued by the farnesyltransferase inhibitor Tipifarnib

**DOI:** 10.1038/s41598-025-91592-x

**Published:** 2025-02-26

**Authors:** Yannik Andrasch, Moses Munene Ireri, Jonas Gander, Ann-Engelke Sabrina Timm, Saravanakkumar Chennappan, Miray Fidan, Melanie Engler, Ion Cristian Cirstea

**Affiliations:** 1 Institute of Comparative Molecular Endocrinology, Ulm University, Ulm, Germany; 2 Masonic Medical Research Institute, Utica, NY USA; 3 Institute of Applied Physiology, Ulm University, Ulm, Germany

**Keywords:** HRAS mutations, Costello syndrome, Osteogenic differentiation, Osteopontin, Tipifarnib, Osteoporosis, Oncogenes, Mechanisms of disease

## Abstract

HRAS is a ubiquitously expressed protein and functions as a central regulator of cellular homeostasis. In somatic cells, mutations in this gene cause cancer, while germline mutations trigger a developmental disorder known as Costello syndrome (CS). Among numerous pathologies, adult CS patients develop osteoporosis. Previous studies revealed that HRAS is implicated in bone homeostasis by controlling osteoblast differentiation, adaptation to mechanical strain and repression of RANKL expression in mature osteoblasts, and by regulating osteoclast differentiation. However, the impact of HRAS on osteoblast differentiation is still debatable. In this study, we created stable doxycycline inducible cell lines overexpressing HRAS G12 mutants in MC3T3-E1 preosteoblast cell line and analyzed their impact on osteoblast differentiation. We demonstrated an inhibitory role of HRAS G12S and HRAS G12V mutants on osteogenic differentiation and identified an increased expression of Opn in an HRAS-dependent manner, which directly correlated with impaired osteogenesis, and was rescued by the farnesyl transferase inhibitor Tipifarnib. At the molecular level, Tipifarnib was not able to block HRAS activation, but impaired HRAS localization to the plasma membrane, and inhibited MAPK activation and Opn expression. Thus, HRAS abundance/activation and its potential crosstalk with OPN may be more critical for osteogenic differentiation than previously assumed.

**Supplementary Information:**

The online version contains supplementary material available at 10.1038/s41598-025-91592-x.

## Introduction

Bone remodeling is a continuous and dynamic process that couples bone formation and resorption, and is essential to prevent accumulation of bone damage to preserve mechanical strength and functionality throughout life^1^. This remodeling process is controlled by molecular mechanisms that include not only differentiation, maturation and activity of osteoblasts (OBs), osteocytes and osteoclasts (OCs), but also their cross-regulation^2^. OBs primarily function as bone forming cells and originate from mesenchymal stem cells (MSCs). OB differentiation and mineralization is orchestrated by various key signaling molecules, such as bone morphogenetic proteins (BMPs), transforming growth factor-beta (TGF-β), fibroblast growth factors (FGFs) and WNT signaling, as well as transcription factors such as runt-related transcription factor 2 (RUNX2) and Osterix (SP7)^3,4^. During OB maturation, cells synthesize and secrete extracellular matrix components including the type I collagen, osteopontin (OPN), osteocalcin, and other non-collagenous proteins, which provide a scaffold and are mineralized by hydroxyapatite crystals^5^. Therefore, a misbalance in bone forming cells is a major contributor to skeletal defects such as osteopetrosis, osteopenia and osteoporosis, which are a major health concern for elderly and people with genetic predispositions^6^.

Among various pathological features, patients diagnosed with developmental disorders named RASopathies were described with a high prevalence of skeletal disorders^7,8^. RASopathies are triggered by mutations in gene encoding for components of the rat sarcoma (RAS)-mitogen activated protein kinase (MAPK) signaling axis^9^. RAS proteins are small GDP/GTP-binding proteins that act as molecular switches, thereby regulating numerous signaling pathways and biological processes such as proliferation, differentiation, and apoptosis, to name only a few. RAS somatic mutations hotspots are within codons 12, 13, and 61 and are associated with more than 30% of human cancers^10^, while germline mutations are associated with the aforementioned RASopathies. HRAS (Harvey RAS) germline mutations cause Costello syndrome (CS)^11^ and the vast majority of mutations affect the oncogenic codon 12, with > 80% of CS patients harboring a glycine 12 substitution to serine (G12S)^11–13^. Glycine 12 substitution to valine (G12V) is a rarer mutation and induces a severe and early lethal pathophenotype, presumably due to higher levels of the GTP-bound active form of HRAS G12V than those of HRAS G12S^14,15^. The adult phenotype of CS has striking features of premature aging, such as aged facial and skin appearance, hair loss, postural deficit, reduced muscle strength, increased incidence of benign and malignant tumors, and osteopenia and osteoporosis^11,16,17^.

Several studies have highlighted the essential role of the RAS-MAPK and -phosphoinositide 3 kinase (PI3K)-AKT axes for proper bone remodeling through the regulation of osteoblasts and osteoclasts homeostasis^18–23^. RAS-extracellular signal-regulated kinase 1/2 (ERK1/2) activation is implicated in the regulation of the central master regulator RUNX2, that induces osteoblast-specific gene expression in early steps of differentiation^24^. Overactivation of KRAS signaling in immature osteoprogenitor cells induces increased proliferation of descendant stromal cells and subsequently increased bone mass, mediated by ERK1/2 and PI3K pathways^25^. In addition, the loss of the negative regulator of RAS neurofibromatosis type 1 (NF1) in mouse osteoblast progenitor cells led to reduced bone mineralization^26^. Among RAS family members, HRAS is the RAS family member that is strongly associated with the regulation of osteoblast homeostasis in response to mechanical strain, which leads to increased levels of active HRAS and induces the expression of RANKL, thus triggering osteoclast differentiation and bone remodeling^27^. Another study identified that in human osteoblasts grown from metaphyseal trabecular bone explants collected from osteoarthritis patients, overexpression of HRAS G12V reduces adhesion and induces apoptosis^28,29^. Furthermore, the HRAS G12S mutation inhibited osteogenesis in a RUNX2-independent manner and altered the expression of extracellular matrix (ECM) remodeling proteins in osteoblasts differentiated from CS patient-derived induced pluripotent stem cells (iPSC)^28^. In contrast, our group recently reported that bone loss in a CS mouse model harboring the *Hras G12V* germline mutation was driven by increased osteoclastogenesis in vitro, while mutations did not alter bone mineral density, osteoblast number quantified on ex vivo bone sections and osteoblast differentiation from bone marrow stromal cells in vitro^22^. However, since previous reports observed mutant HRAS-mediated alterations in OBs^28,29^ one can speculate that in the Hras G12V mouse model either Hras expression and activation did not reach inhibitory levels or an unknown mechanism safeguards osteoblast differentiation. Last but not least, a recent study in a mouse model of cutaneous skeletal hypophosphatemia syndrome model revealed that Hras mutant expressed in an inducible manner in the appendicular skeleton led to a reduction in bone mass and mineralization, periosteal osteosarcoma-like lesions, elevated levels of plasma active FGF23, hypophosphatemia and phosphaturia. Unfortunately, the quantification of osteoblasts on bone sections and in vitro differentiation to identify the impact of Hras G12V mutant was not performed^30^.

Here, we tested whether the overexpression of two CS mutations, *HRAS G12S* and *G12V*, respectively, affect differentiation of murine MC3T3-E1 preosteoblasts and whether we are able to rescue it using inhibitors of proteins associated with RAS signaling. We identified that both HRAS G12 mutations inhibited osteoblast differentiation, with G12V mutations having a stronger inhibitory effect. At the molecular level, both mutants inhibited the expression of master regulators Runx2 and increased expression of Opn. As a proof of concept, using a farnesyltransferase inhibitor, Tipifarnib, we rescued osteoblast differentiation in both HRAS G12 mutants and we successfully reversed impairments in mineral deposition in response to overexpression in WT and G12S, but only partially in G12V expressing cells.

## Results

### Overexpression of recombinant HRAS G12S and HRAS G12V mutants leads to impaired osteoblast differentiation in MC3T3-E1 preosteoblast cell line

Using a CS mouse model, our group recently demonstrated that the *Hras G12V* point mutation led to bone loss, triggered by an MAPK- and PI3K-signaling dependent increased osteoclastogenesis. However, no effect was observed on osteoblast differentiation from bone marrow stromal cells in these mice^22^, while recent data revealed that *HRAS G12S* impaired differentiation of osteoblast differentiation obtained from CS patient-derived iPSC^28^. Therefore, we aimed to study the effect of inducible overexpression of G12V and G12S mutations on in vitro osteoblast differentiation in the murine preosteoblast cell line MC3T3-E1.

**Fig. 1:**
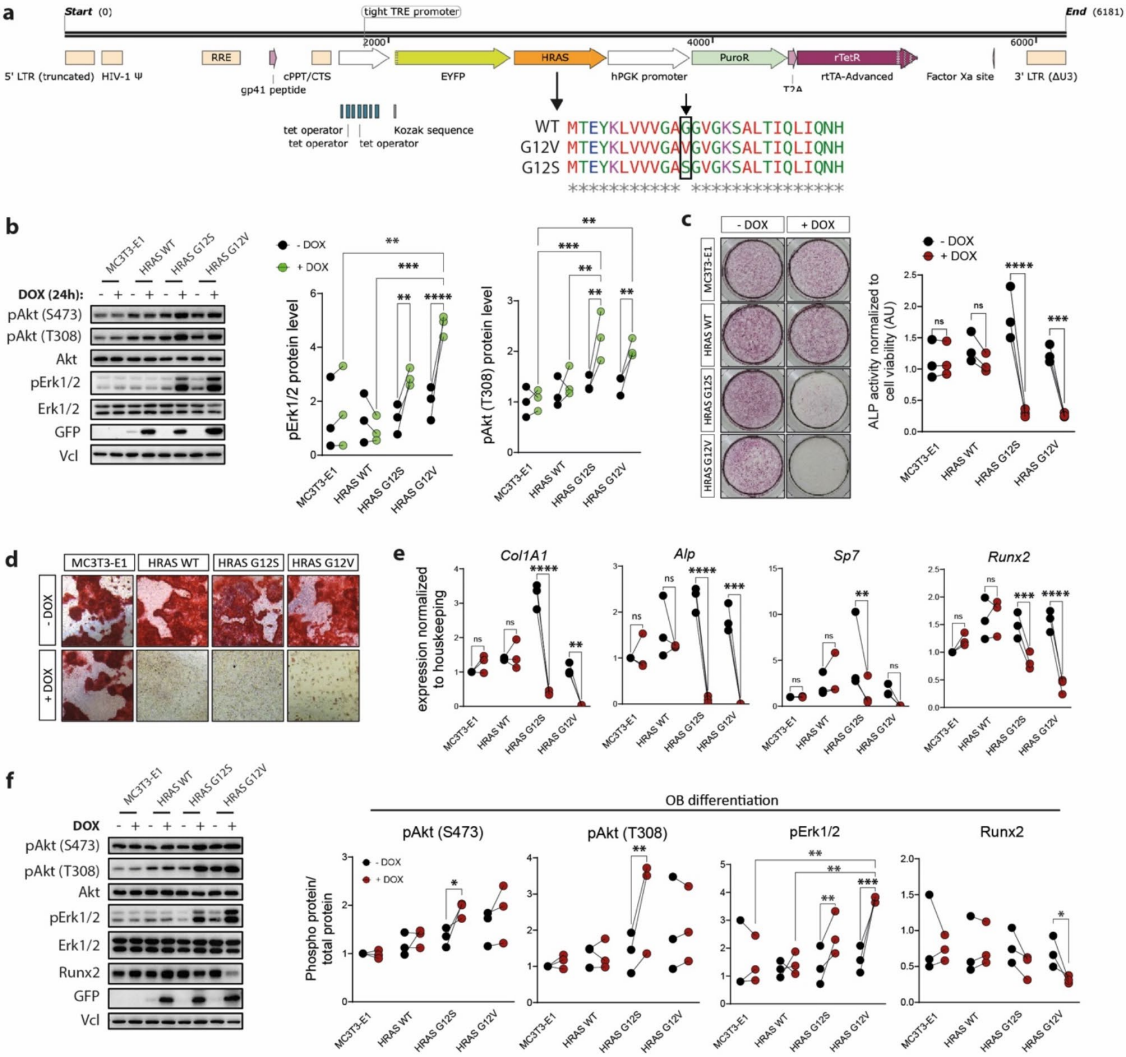
Overexpression of recombinant HRAS G12S and G12V mutants impaired osteoblast differentiation in MC3T3-E1 preosteoblast cell line. (**a**) Architecture of the lentivirus integration cassette harboring either HRAS wild type (WT) or G12S or G12V mutants (created with SnapGene Viewer). (**b**) The effect of recombinant HRAS WT, G12S and G12V on downstream signaling was analyzed 24 h after doxycycline (DOX) induction. Activation of MAPK and Akt pathways was analyzed by immunoblotting with specific antibodies for activating phosphorylations of Erk1/2 and Akt (Ser473 and Thr308). Total Akt and Erk1/2 blots were used as control for their phosphorylated-forms, whereas vinculin (Vcl) and GFP served as loading and recombinant protein expression controls, respectively. Immunoblot signals were quantified using BioRad ImageLab software (*n* = 3 independent experiments). Original blots are presented in Supplementary Fig. 3. (**c**–**f**) MC3T3-E1 and -overexpressing recombinant HRAS were differentiated into osteoblasts using osteogenic induction media. (**c**) Qualitative and quantitative assessment of osteoblast differentiation 7 days after osteogenic induction. Alp activity was measured using Alp assay kit and normalized to cell viability measured by Presto Blue assay kit (*n* = 3). Qualitative assessment was performed by imaging 24-well plates with Canon Sigma DG macro camera. (**d**) Qualitative assessment of mineralization by Alizarin Red S staining was performed 28 days after the induction of osteoblast differentiation (*n* = 3) using Leica DMI6000 microscope at 10x magnification. (**e**) mRNA expression of osteoblast differentiation marker genes *Runx2*, *Sp7*, *Alp*, and *Col1a1* 7 days after osteogenic induction (*n* = 3, each in triplicates). (**f**) Analyses of MAPK and Akt activation, and the expression of Runx2 osteoblast differentiation marker by immunoblotting 7 days after osteogenic induction with specific antibodies for activating phosphorylations of Erk1/2, Akt (Ser473 and Thr308) and osteoblast marker Runx2. Akt and Erk1/2 immunoblots were used as control for their phospho-forms, whereas vinculin (Vcl) and GFP served as loading and recombinant protein expression controls, respectively. Original blots are presented in Supplementary Fig. 4. Immunoblot signals from *n* = 3 independent experiments were quantified using BioRad Image Lab software. For quantified data, plots and statistical analyses were performed using GraphPad Prism 10. Data are represented as single data points of *n* = 3 independent experiments. Asterisks indicate a statistically significant difference from controls (*P* < 0.05, two-way ANOVA).

Using lentiviral transduction, we generated doxycycline (DOX) inducible MC3T3-E1 cells stably overexpressing HRAS WT, and G12S and G12V mutants, fused N-terminally to an *EYFP *reporter gene (Fig. 1A). We performed a preliminary screen to identify clones with comparable EYFP-HRAS expression between WT and mutants (Supplementary Fig. 1A). To exclude off-target effects by the genomic insertion site of the expression cassettes, the inhibitory effects on osteoblast differentiation in an uninduced state (Supplementary Fig. 1B) and impairment of cell viability (Supplementary Fig. 1C) were analyzed. Immunoblotting analysis of selected clones showed equal expression of recombinant EYFP-HRAS fusion proteins after 24 h of DOX induction (Fig. 1B) and DOX induction of both mutants led to a significant increase in activating phosphorylation of Erk1/2 and Akt (Thr308) when compared to uninduced cells (Fig. 1B).

To evaluate osteogenic function, parental MC3T3-E1 cells and HRAS clones were cultured for up to 28 days in osteogenic induction media, containing L-ascorbic acid and β-glycerophosphate. In the absence of recombinant HRAS expression (-DOX), all tested cell lines displayed a high level of alkaline phosphatase (Alp) activity on day 7 of OB differentiation (Fig. 1C). In contrast to parental MC3T3-E1 cells and HRAS WT clone, DOX induction significantly impaired Alp activity in HRAS G12S and HRAS G12V clones (Fig. 1C). Further assessment of osteogenic mineral deposition showed that in contrast to parental cells and uninduced HRAS clones, mineral deposition in clones overexpressing both HRAS mutants was virtually absent under DOX induction. Surprisingly, clones overexpressing HRAS WT also showed decreased mineralization, indicating that although WT can be inactivated and properly regulated, increased level of WT above a certain threshold could become detrimental to late osteogenic stages (Fig. 1D). These data further supported observations of an impaired capability of MC3T3-E1 cells to differentiate towards the osteoblast lineage when HRAS and its downstream signaling activation were enhanced. In addition, gene expression levels of alkaline phosphatase (*Alp)*, type 1 collagen A1 *(Col1A1)*, Osterix (*Sp7)* and Runt-related transcription factors 2 *(Runx2)* were assessed at day 7 of differentiation. RT-qPCR analyses showed a significant reduction in the expression of *Alp*,* Col1A1*, and *Runx2* in response to induction of HRAS G12S and HRAS G12V expression when compared to the uninduced state, whereas *Sp7* was significantly downregulated only in the HRAS G12S clone (Fig. 1E). Since osteoblast differentiation is a tightly regulated process and two major RAS-controlled pathways, MAPK and PI3K respectively, are implicated in this process^19^, we completed our analysis by evaluating their activation on day 7 of OB differentiation by immunoblotting. The HRAS G12S clone showed significantly increased levels of activating phosphorylation of Akt at Serine 473 (S473) and threonine 308 (T308) in response to DOX (Fig. 1F). Furthermore, levels of activating phosphorylations of Erk1/2 were significantly increased in HRAS G12S and HRAS G12V clones after DOX induction when compared to uninduced control samples (Fig. 1F). Neither in the parental MC3T3-E1 cells nor in the HRAS WT cells, an increased activation of both pathways was not observed following DOX induction (Fig. 1F). Finally, protein levels of the osteoblast marker Runx2 were assessed and revealed a significant reduction in the DOX-induced HRAS G12V cells when compared to the uninduced control at day 7 of osteoblast differentiation (Fig. 1F). Taken together, these results suggest that a constitutive activation of HRAS by point-mutations at codon 12 leads to an impaired differentiation of osteoblast precursor cells, with more severe effects in response to G12V mutation.

### Farnesyltransferase (FTase) inhibitor rescues impaired osteoblast differentiation driven by mutant HRAS expression

**Fig. 2:**
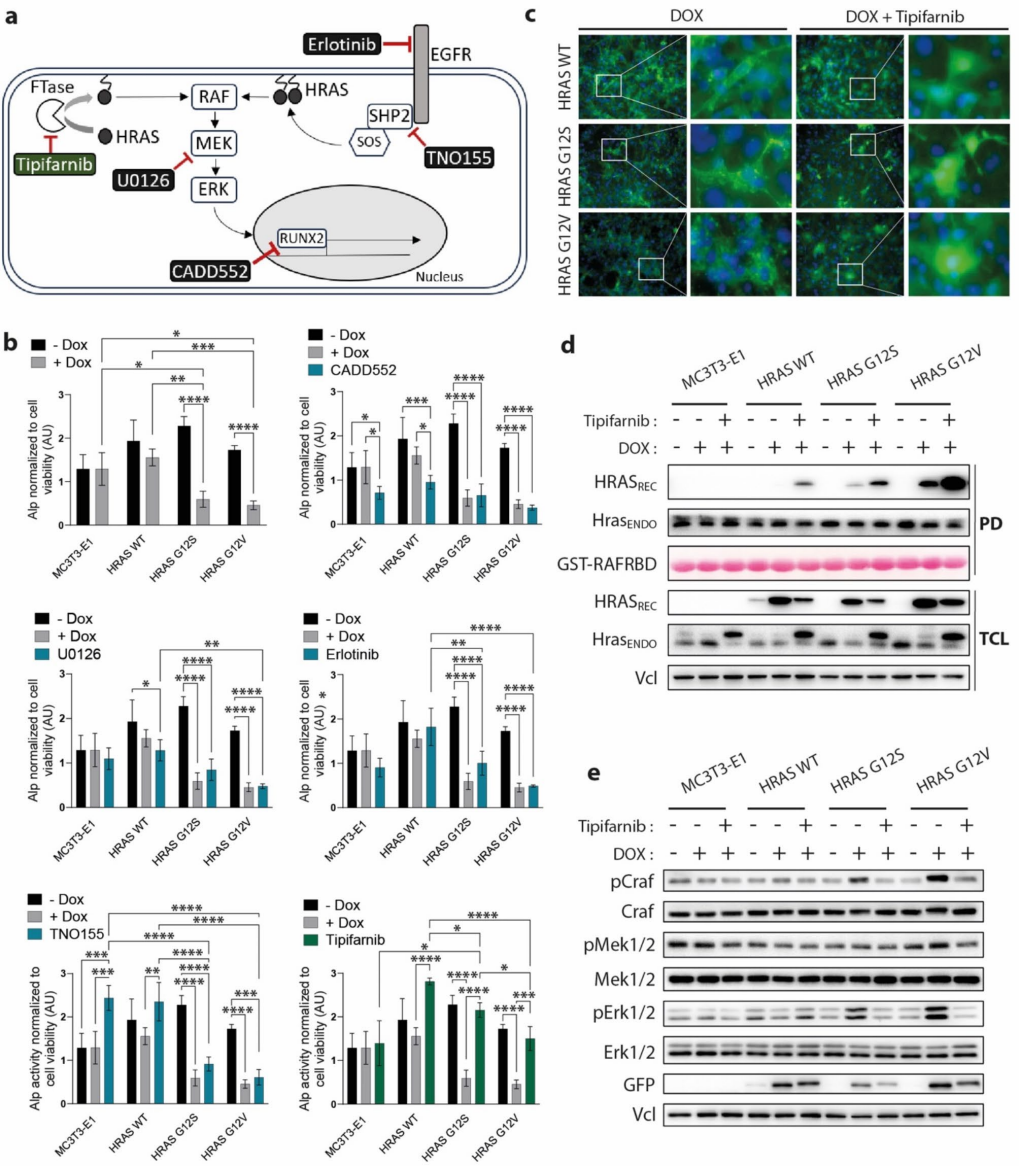
Farnesyl transferase (FTase) inhibitor Tipifarnib rescued early osteoblast differentiation by blocking HRAS membrane localization and downstream activation of MAPK. (**a**) Schematic representation of signaling molecules that were targeted with inhibitors during osteoblast differentiation. (**b**) Differentiation of MC3T3-E1 cells in response to DOX and inhibitors treatment: FTase inhibitor (Tipifarnib; 0.5 µM), RUNX2 inhibitor (CADD522; 50 µM), SHP2 inhibitor (TNO155; 1 µM), MEK1/2 inhibitor (U0126; 10 µM) or EGFR inhibitor (erlotinib; 5 µM). Alp activity was measured 7 days post osteogenic induction using Alp assay kit and was normalized to cell viability measured by Presto Blue assay kit (*n* = 3 independent experiments). (**c**) Microscopy analysis of HRAS localization in cells treated with Tipifarnib (0.5 µM) for 24 h. EYFP-HRAS fusion protein was visualized by immunofluorescence microscopy at 488 nm using Leica DMI6000B at 20x magnification. Nuclei were stained using DAPI. (**d**, **e**) Immunoblotting analyses of MC3T3-E1 subclones in the presence or absence of DOX and Tipifarnib (0.5 µM) after 24 h. (**d**) Activation of endogenous Hras (Hras_ENDO_) and recombinant HRAS (HRAS_REC_) was monitored by pull down (PD) assay using as bait the RAS-binding domain (RBD) of RAF1 kinase coupled to glutathione-S-tranferase (GST), GST-RAF1-RBD. PD samples were analyzed by immunoblotting for HRAS activation using HRAS antibody, while immunoblots using same antibody in total cell lysates (TCLs) were used as controls to monitor HRAS_REC_ and Hras_ENDO_ protein abundance. Original blots are presented in Supplementary Fig. 5. (**e**) Craf-Mek1/2-Erk1/2 activation was monitored with specific antibodies for activating phosphorylations of Craf, Mek1/2 and Erk1/2. Craf, Mek1/2 and Erk1/2 immunoblots were used as control for their phospho-forms, whereas vinculin (Vcl) and GFP served as loading and recombinant protein expression controls, respectively. Original blots are presented in Supplementary Fig. 5.

Having identified that HRAS G12 mutations lead to increased downstream signaling and impaired differentiation of MC3T3-E1 preosteoblasts, we next aimed to rescue osteoblast differentiation in the HRAS G12S and HRAS G12V clones, using commercially available inhibitors. Classical inhibitors directly targeting the EGFR-RAS-MAPK signaling axis, including EGFR (erlotinib), SHP2 (TNO155) and MEK1/2 (U0126), but also indirect inhibition of HRAS activation using the CAAX-competitive inhibitor of farnesyltransferase (FTase), Tipifarnib were used^31^. As control, we included CADD552, a specific inhibitor of RUNX2 (Fig. 2A). The impact of these inhibitors on osteoblast differentiation was analyzed by measuring Alp activity after 7 days of osteogenic differentiation (Fig. 2B and Supplementary Fig. 2A). As expected, the RUNX2 inhibitor CAD552 significantly reduced Alp activity and osteoblast differentiation in all tested cells. While the EGFR-MAPK targeting inhibitors erlotinib, U0126 and TNO155 were not able to rescue osteoblast differentiation in the HRAS G12V and G12S clones, the FTase inhibitor Tipifarnib was able to rescue differentiation to the level of the uninduced control samples (Fig. 2B and Supplementary Fig. 2). Furthermore, in contrast to other tested inhibitors, Tipifarnib did not affect cell viability (Supplementary Fig. 2B).

**Fig. 3:**
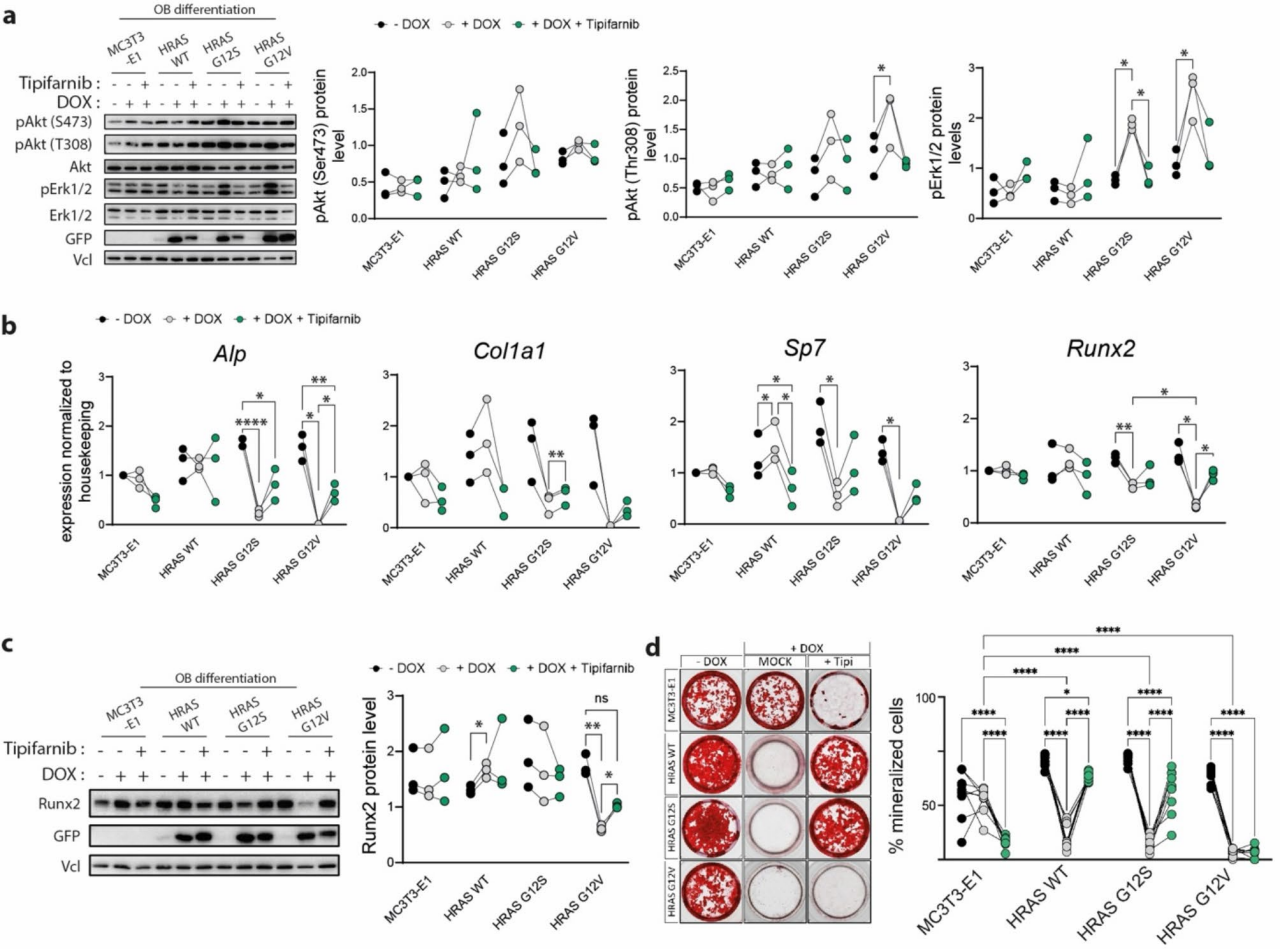
Tipifarnib rescued osteoblast differentiation in cell lines expressing recombinant HRAS mutants. (**a**) Schematic representation of MAPK pathway and inhibitors used in this study (created using Adobe Illustrator). (**b**–**c**) MC3T3-E1 cells kept under osteogenic induction for 7 days, in the presence or absence of DOX and Tipifarnib (0.5 µM). Immunoblotting analyses of Erk1/2 and Akt activation were performed with specific antibodies for activating phosphorylations of Erk1/2, Akt (Ser473 and Thr308, respectively). Akt and Erk1/2 immunoblots were used as control for their phospho-forms, whereas Vcl serves as loading control and GFP as control for expression of recombinant protein. Original blots are presented in Supplementary Fig. 6. Signal intensity of 3 independent experiments was quantified using BioRad Image Lab software, normalized to loading control and total protein abundance, respectively. (**b**) Expression of osteoblast specific markers *Runx2*,* Sp7*,* Alp* and *Col1a1* relative to housekeeping genes was measured by qRT-PCR (*n* = 3). (**c**) Immunoblotting analyses of Runx2 expression, whereas vinculin (Vcl) and GFP served as loading and recombinant protein expression controls, respectively. Immunoblot signals from *n* = 3 independent experiments were quantified using BioRad Image Lab software. Original blots are presented in Supplementary Fig. 7. (**a**–**c**) Statistical analyses were performed using GraphPad Prism 10. Data are represented as single data points of *n* = 3 independent experiments. Asterisks indicate a statistically significant difference from controls (*P* < 0.05, two-way ANOVA). (**d**) Assessment of mineralization by Alizarin Red S staining was performed 27 days after osteogenic and DOX induction, and in the presence or absence of Tipifarnib (0.5 µM). The percentage of the well area covered with mineralized extracellular matrix was calculated using ImageJ (*n* = 9 independent experiments).

FTases catalyze post-translational attachment of farnesyl groups needed for localization and activation of HRAS at the membrane^32^, the subcellular site where RAS binds to and activates effector proteins such as CRAF^33,34^. As proof of principle, we monitored the subcellular localization of EYFP-HRAS in response to Tipifarnib by fluorescence microscopy and observed that HRAS relocated from membrane clusters to cytoplasmic compartment, irrespective of HRAS mutation status (Fig. 2C). Immunoblotting data in total cell lysates showed that Tipifarnib treatment led to an increased abundance of the non-farnesylated forms, for both endogenous and recombinant HRAS, demonstrated by an electrophoretic mobility shift between non-treated and treated samples (Fig. 2D), where non-farnesylated Hras displayed slower mobility in SDS-PAGE^35^. Interestingly, the amount of recombinant HRAS in the total cell lysates was reduced in Tipifarnib treated cells. Due to the fact that active GTP-bound RAS resides at the plasma membrane and that inactive HRAS relocates to the cytoplasm, we monitored the activation state of HRAS in response to Tipifarnib treatment by pulldown assay using the RAS binding domain of RAF1 (RAF1-RBD) fused to glutathione S-transferase (GST) as a bait. In contrast to a reduction of total recombinant protein, activation of recombinant HRAS was increased in response to Tipifarnib (Fig. 2D). Furthermore, increased activation of HRAS did not translate into an increased activation of the CRAF-MAPK downstream pathway, monitored by immunoblotting with phospho-specific antibodies for their active forms in undifferentiated MC3T3-E1 clones (Fig. 2E). Next, we analyzed the impact of Tipifarnib on downstream signaling in differentiating cells 7 days after osteogenic induction and observed that treatment reduced downstream activation, especially activation of Erk1/2, in the HRAS G12S- and G12V-expressing cells (Fig. 3A). We evaluated the impact of Tipifarnib on the expression of osteoblast differentiation markers *Alp*, *Col1A1*, *Sp7* and *Runx2* in the presence or absence of DOX and saw that the expression of *Alp*,* Sp7*, and *Runx2* in DOX-induced HRAS G12S and HRAS G12V clones was significantly reduced when compared to the uninduced state and partly rescued by Tipifarnib (Fig. 3B). Furthermore, immunoblotting analysis showed that the reduction of Runx2 expression upon DOX induction reached statistical significance only for G12V mutant, whose expression was partly rescued by Tipifarnib treatment (Fig. 3C). To further validate the efficiency of Tipifarnib to rescue osteoblast differentiation triggered by HRAS G12V and HRAS G12S mutants overexpression, calcified matrix deposition was analyzed on day 28 of osteogenic induction by Alizarin Red S staining. While Tipifarnib was able to rescue osteogenic mineral deposition in the HRAS WT and G12S cells, it failed to improve osteogenic mineral deposition in the HRAS G12V clone (Fig. 3D). Interestingly, in the parental MC3T3-E1 cells, the treatment with Tipifarnib led to a significantly reduced deposition of calcified matrix (Fig. 3D), further highlighting a potential role of RAS as a notable regulator of osteoblast differentiation. These results not only demonstrate that HRAS plays an important role during osteoblast differentiation through its interaction with effectors at the plasma membrane, but also further proves that HRAS G12V mutation has a more detrimental impact on osteogenic differentiation than G12S mutation.

### Expression of HRAS G12S and G12V mutants enhances Opn expression

**Fig. 4:**
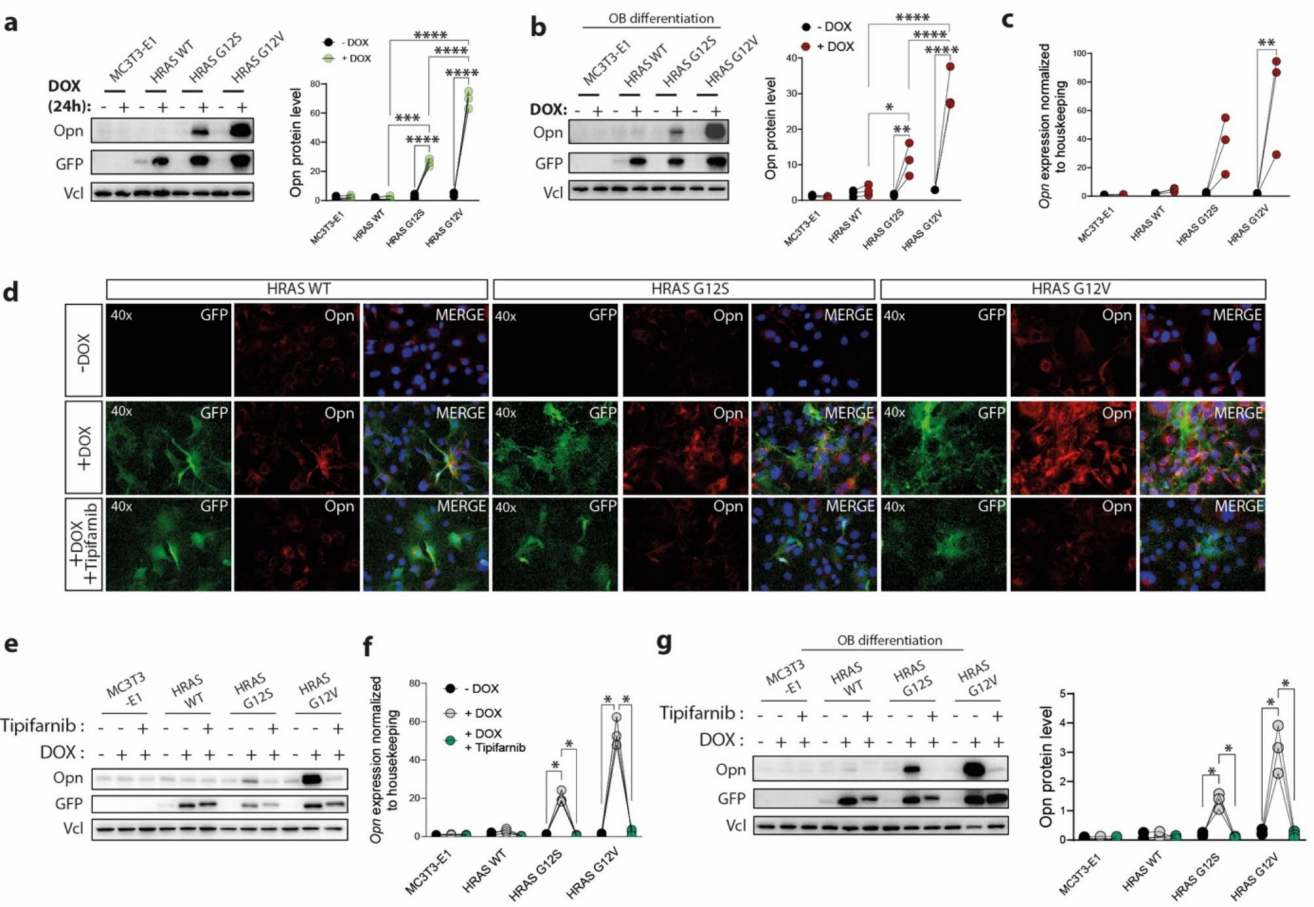
Overexpression of HRAS G12S and G12V mutants correlated with an increased expression of osteopontin (Opn). (**a**, **b**) Protein expression of Opn was monitored 24 h postinduction with DOX (**a**) and 7 days after osteogenic induction (**b**) by immunoblotting using specific antibodies. Vinculin (Vcl) and GFP served as loading and recombinant protein expression controls, respectively (*n* = 3 independent experiments). Original blots are presented in Supplementary Figs. 8–9. Quantification of immunoblot signals was done using BioRad Image Lab software. (**c**) mRNA expression of *Opn* was measured using qRT-PCR after 7 days of DOX and osteogenic induction (*n* = 3). (**d**, **e**) Opn protein expression in response to tipifarnib treatment for 24 h in HRAS WT, HRAS G12S and HRAS G12V expressing MC3T3-E1 cells, (**d**) analyzed by immunofluorescence staining using Opn primary antibody and secondary antibody coupled to AlexaFluor 594 (red). HRAS coupled to EYFP (green) was used as control for recombinant HRAS protein expression, while nuclei are counterstained with DAPI (blue). (**e**) Opn protein levels were monitored by immunoblotting using specific antibody. Vinculin (Vcl) and GFP served as loading and recombinant protein expression controls, respectively. (**f**) mRNA expression of *Opn* as measured using qRT-PCR, 7 days after DOX and osteogenic induction in the presence or absence of tipifarnib (0.5 µM) (*n* = 3). (**g**) Immunoblotting analyses of Opn expression in MC3T3-E1 cells kept under osteogenic induction for 7 days, in the presence or absence of DOX and Tipifarnib (0.5 µM), using specific antibodies. Vinculin (Vcl) and GFP served as loading and recombinant protein expression controls, respectively (*n* = 3 independent experiments). Original blots are presented in Supplementary Fig. 10. Quantification of immunoblot signals was done using BioRad ImageLab software. Uncropped blots are displayed in *Supplementary information*.

Previous studies on the impact on bone homeostasis of RAS negative regulator NF1 and RAS downstream effector BRAF indicated that OPN expression is controlled by the RAS-MAPK pathway and increased OPN expression is associated with reduced osteogenic differentiation^36,37^. Opn is generally a marker for osteogenic differentiation and a major non-collagenous protein in the extracellular matrix of mineralized tissue^38^. It is highly expressed in bone lesions and rheumatoid arthritis^39^, and promotes malignant cell proliferation, detachment, invasion, and metastasis of several osteotropic cancers^40^. We monitored Opn expression in preosteoblast cell line by immunoblotting and we observed that the DOX-induced expression of HRAS G12S and HRAS G12V led to a significant increase of Opn protein levels when compared to parental cell line, HRAS WT clone and uninduced mutant clones (Fig. 4A). After osteogenic induction, we detected similarly increased levels of Opn transcript and protein in HRAS G12S and HRAS G12V clones (Fig. 4B, C) and G12V mutant overexpression promoted higher expression of Opn than G12S, which correlated with a stronger downstream signaling activation and stronger Runx2 inhibition in G12V cells (Fig. 1F A–C). Having observed that Tipifarnib was able to rescue osteoblast differentiation in clones expressing HRAS G12S and HRAS G12V (Figs. 2B and 3D), we then aimed to see whether Tipifarnib has an impact of Opn levels in treated cells. Opn transcript and protein levels were analyzed and after 24 h of treatment, Opn expression in HRAS G12V and HRAS G12S DOX-induced clones was reduced to the level of uninduced samples (Fig. 4D–F). Moreover, this effect was specific for Tipifarnib and was not observed in response to any other tested inhibitors (Supplementary Fig. 2C). Finally, Opn levels on day 7 in HRAS G12S and HRAS G12V differentiating osteoblast treated with Tipifarnib were also significantly reduced (Fig. 4G). These results demonstrated that the expression and activation of HRAS mutants at the plasma membrane induces Opn expression in the preosteoblasts and osteoblasts and that HRAS mutants impaired osteoblast differentiation and mineralization correlated with increased Opn expression. Therefore, Opn may well be a promising target to treat not only skeletal disorders arising from HRAS germline mutations in CS patients, but also other skeletal pathologies associated with increased RAS activation in RASopathies and other diseases.

## Discussion

HRAS G12 mutations associated with CS and cutaneous skeletal hypophosphatemia syndrome (CSSH) trigger bone loss^8,30^. Data obtained from mouse models of the above-mentioned diseases indicated that bone loss occurred due to an increased osteoclastogenesis^22^ and reduced phosphate and impaired mineralization, respectively^30^. However, the latter study failed to provide information on whether mutations affected osteoblast number in CSSH. Thus far, the impact of HRAS on osteoblast differentiation in vitro and in vivo models is controversial and depends on the research model used for studies. In vitro differentiation of CS HRAS G12S iPSCs into osteoblasts is impaired, while our previous study using CS Hras G12V mouse bone marrow stromal cells indicated that osteoblast differentiation is not affected^22,28^. This discrepancy may be due to differences between human iPSCs and CS mouse primary cells, where iPSCs are uncoupled from niche factors and interactions with other cell types and may therefore undergo a detrimental differentiation route that is not balanced by bone marrow niche interactions. Our current in vitro study indicated that HRAS G12V mutant overexpression is indeed detrimental for osteoblast differentiation. Considering this, in the CS Hras G12V mouse model, either niche interactions are critical for preserving osteoblast differentiation or a critical threshold of Hras G12V expression and activation has to be reached in order to impair osteoblast differentiation.

In contrast to CS HRAS G12S iPSCs differentiation^28^, our study identified a Runx2-dependent mechanism as a result of an increased MAPK activation. Our data is supported by a previous publication from the same group, which showed an impaired in vitro osteogenesis of iPSCs created from cardio-facio-cutaneous syndrome (CFCS) RASopathy patient cells carrying activating mutation BRAF Q257R, as a result of increased ERK1/2 activation and a reduced RUNX2 expression^37^. Moreover, we also revealed that G12V mutant is a stronger inhibitor of Runx2 expression than G12S, which also correlated with a stronger inhibition of osteogenesis, further proving observations from cancer studies data and clinical observations from CS patients^14,41^. It is well established that ERK1/2 is a major regulator of RUNX2 through direct binding and activating phosphorylations, leading to increased transcriptional activities that positively affect differentiation^42–45^. These discrepancies may well be related to the upstream signals that engage ERK1/2-MAPK-RUNX2 signaling, where FGF-FGFR-ERK1/2 does not control RUNX2 expression^46^, while activation of EGF/EGFR/ERK1/2 inhibited the expression of RUNX2^47^. Similar to our observations, literature data indicated that either EGFR activation or a hyperactivation of ERK1/2 by a constitutive activation of MEK1 inhibited osteoblast differentiation in a RUNX2-depedent manner^47,48^, denoting EGFR-HRAS-ERK1/2 signaling axis as a major regulator of RUNX2-dependent osteoblast differentiation. Last, but not least, as RUNX2 also has a tumor suppressive function^49^, we can speculate that a reduction of RUNX2 may well be an adaptative response of HRAS oncogenic signaling to repeal RUNX2 effects on proliferation, thus becoming detrimental for osteoblast differentiation.

In our study, HRAS mutants enhanced OPN expression in preosteoblasts and differentiated osteoblasts. OPN is a multifunctional extracellular matrix glycoprotein and in bone is produced by mature OBs and either stimulates^50–52^ or inhibits osteoblastic differentiation, mineralization and bone formation^53,54^. Increased levels of OPN are considered as a marker for bone disorders such as defective mineralization^55^, rheumatoid arthritis^56^ and bone metastasis^57^. An increased expression of OPN in response to our HRAS mutants analysis was also reported in a study of the differentiation of CFCS BRAF Q257R iPSCs^37^ and in the heterozygous loss of the RAS negative regulator neurofibromin 1 (NF1) in osteoblasts, correlated with impaired osteoblasts differentiation and mineralization^36^, indicating that a balanced HRAS activation/OPN expression may well be critical for proper osteogenesis. Also, osteoblast-derived Opn promoted increased osteoclast migration and bone resorption in the Nf1 +/- mouse model of neurofibromatosis type 1 (NF1) syndrome^58^. However, OPN knockdown in vitro differentiated CFCS BRAF Q257R iPSCs did not improve differentiation and mineralization, indicating that multiple RAS-mediated pathways may be implicated. Interestingly, the activation of p90RSK by the RAS-ERK1/2 increases the expression of ligand/receptor autocrine loop involving OPN/CD44^59^. OPN/CD44 functions as an activator of RAS-MAPK pathway^60^, thus initiating a feed forward loop suppressing osteogenesis. As OPN may well be an important factor that contributes to osteopenia and osteoporosis in CS and other RASopathies, future studies may address yet unknown OPN-related properties that would contribute to bone health such as its phosphorylation status, amount of soluble OPN that may positively stimulate osteoclastogenesis, osteoblast responsiveness to growth factors^61^, and activation of CD44 and/or integrins^62^. Last, but not least, future studies have to focus on the identification of the stage at which an enhanced activation of HRAS and an impaired HRAS-OPN cross-talk becomes detrimental for the osteoblast differentiation axis mesenchymal stem cell-preosteoblast-osteoblast.

Inhibition of MAPK activation by MEK inhibitors was successfully used in various in vivo and in vitro research models of RASopathy pathophenotype related to prenatal lethality, craniofacial abnormalities, cardiac defects, skeletal myopathy, and enhanced osteoclastogenesis^22,63,64^. Additionally, in an in vitro model for osteoblast differentiation, Choi and colleagues were able to rescue the impaired differentiation, but not mineralization, using MEK inhibitor in CS iPSCs^28^. In contrast, in our set up, U0126 treatment only partially rescued G12S differentiation, but had no effect on G12V differentiation. These differences remain unclear and may well be related to differences in cellular models, HRAS mutant strength, and their expression and activation levels. The most successful rescue attempt used in our study was with FTI Tipifarnib, which was able to completely rescue osteogenesis in HRAS G12S cells, while in G12V cells was able to rescue only differentiation, but not mineralization. Another FTI, FTI-277 was reported to inhibit osteogenic differentiation and mineralization of human mesenchymal stem cells^65^, therefore we assume that Tipifarnib treatment tuned down cellular signaling to a level that rescued osteogenesis in our mutant HRAS cell models. Although Tipifarnib, as seen with other FTIs, can have effects not only on RAS GTPases, but also on other proteins such as HDJ-2, CENPE, PRL^65–67^, Tipifarnib seems to be quite an efficient therapeutical approach against hyperactive HRAS-driven cancers^32,68,69^ and in KRAS-driven cancers in combination with KRAS G12C inhibitors^70^. Moreover, as farnesyl moieties are a product of mevalonate pathway and statins were successfully used in rescue experiments for different pathologies in various animal models of RASopathies^71–74^, Tipifarnib also has the potential to be considered as a treatment strategy for skeletal defects, among potentially others.

Our findings indicated that CS HRAS oncogenic mutations G12S and G12V impaired osteogenesis by affecting osteoblast differentiation and mineralization. At the molecular level, both mutations inhibit Runx2 expression and at the same time enhance Opn expression. The inhibition correlated with mutation strength, proven by the G12V mutation that displayed high levels of active HRAS and triggered more severe effects. Despite using an overexpression system, our data suggest that in a pathological situation that leads to high levels of expressed and active HRAS, osteogenic signaling may be impaired by changes in the Runx2 and Opn expression, and bone pathophenotype in CS and other RASopathies may be amenable to inhibitors of RAS farnesylation.

## Materials and methods

### Cell culture

MC3T3-E1 murine preosteoblast cells were maintained in alpha Minimal Essential Medium (α-MEM; Life Technologies) supplemented with 10% fetal bovine serum (FBS; Gibco) and 1% penicillin/streptomycin (P/S) mixture. For the induction of recombinant protein expression, media was further supplemented with DOX (100 µg/ml).

### Lentivirus production and transduction

pCW-EYFP-HRAS WT, -G12S and -G12V inducible vectors were obtained by ligation of HRAS fragment cut with *Nhe*I and *Bam*HI enzymes into the pCW-EYFP inducible vector, cut with the same enzymes. Clones obtained from the transformation of ligation mixtures were sequenced to verify the accuracy of inserted fragments. Lentiviruses containing the inducible expression cassettes for HRAS WT, HRAS G12S and HRAS G12V coupled to EYFP were produced in HEK293T cells using PEI transfection of HRAS plasmids together with psPAX2 (Addgene #12260) and pMD2.G (Addgene #12259) plasmids. Lentivirus-containing media was harvested after 48 h and 72 h. For each transduction, 10,000 MC3T3-E1 cells were resuspended in 1 ml lentiviral particles-containing DMEM and 8 µg/ml polybrene was added. Cell/polybrene/virus mix was centrifuged for 40 min at 600 rpm at RT. The pellet was resuspended in 100 µl DMEM and plated in a well of a 96-well plate. Selection started after 24 h incubation at 37 °C by changing to medium supplemented with 3 µg/ml puromycin. 100 single cells were seeded in 6-well plates and single colonies were picked after 10 days of incubation in puromycin and further grown in DMEM for expansion and clonal screening.

### Inhibitors screening

For the inhibitor screening, 2.5 × 10^4^ cells were seeded in 24-well plates and cultured for 24 h in a-MEM, 10% FBS, 1% P/S. On the following day, media was changed to osteogenic induction media (α-MEM, 10% FBS, 1% P/S, 100 µg/ml L-Ascorbic acid (Sigma-Aldrich) and 5 mM β-glycerophosphate (Sigma-Aldrich)). The osteogenic induction media was further supplemented with DOX (100 µg/ml) for induction of recombinant protein expression and the indicated inhibitors: RUNX2 inhibitor (CADD522; Selleckchem, #S0790) 50 µM, FTase inhibitor (Tipifarnib; Selleckchem, #S1453) 0.5 µM, SHP2 inhibitor (TNO155; Selleckchem, #S8987) 1 µM, MEK1/2 inhibitor (U0126; Selleckchem, #S1102) 10 µM, PI3K inhibitor (LY294002; Selleckchem, #S1105) 5 µM, EGFR inhibitor (Erlotinib; Selleckchem, #S7786) 5 µM. The media was changed at approximately 72 h intervals throughout the experiment and cells were analyzed on day 7 via ALP assay and immunoblotting. For mineralization assay using Alizarin Red S, cells were analyzed on day 27. Three independent experiments were performed, each with technical replicates.

### Alkaline phosphatase (ALP) activity, Alizarin Red S and PrestoBlue staining

To evaluate cell viability, Alp enzyme activity and mineral deposition, PrestoBlue cell viability reagent (Life Technologies), ALP kit (Sigma-Aldrich), and Alizarin Red S reagent (Sigma-Aldrich), respectively, were used according to the manufacturers’ instructions and as previously described^75^.

### Immunoblotting analyses

Cells were lysed in modified RIPA buffer (1% IGEPAL Ca-630, 10% glycerol, 50 mM Tris, 2 mM MgCl2, 100 mM NaCl, 20 mM β-Glycerophosphate, 1 mM Na3VO4, with freshly added EDTA-free Protease Inhibitor Cocktail [Roche]). Lysed cells were centrifuged at 18,000 x g for 10 min and protein concentrations of the supernatants were determined using the BCA Assay Kit (#23225; Pierce). Proteins were separated by SDS-PAGE in 10% PAA gels under reducing conditions and transferred on to a nitrocellulose membrane (#1620112; Bio-Rad). Membranes were blocked with 5% BSA in TBST for 1 h at room temperature followed by incubation with primary antibodies (anti-ERK1/2 [#9102, 1:1,000; Cell Signaling], anti-pERK1/2 Thr202/Tyr204 [#4370, 1:1,000; Cell Signaling], anti-AKT [#9272, 1:1,000, Cell Signaling], anti-pAKT Ser473 [#4060, 1:1,000, Cell Signaling], anti-pAKT Thr308 [#2965, 1:1,000, Cell Signaling], anti-OPN [#AF808, 1:1,000, R&D], anti-RUNX2 [#12556,1:1,000, Cell Signaling], anti-GFP [#2956, 1:1,000; Cell Signaling], anti-vinculin [#sc-73614, 1:2,000; Santa Cruz]) overnight at 4 °C and 1 h at room temperature with secondary antibodies (horseradish peroxidase-conjugated goat anti-mouse IgG [#P0447; Dako] and anti-rabbit IgG [#A6154; Invitrogen]). Immobilon Forte Western HRP substrate (#WBLUF0500, Millipore) was used for signal detection by the ChemiDoc MP Imaging System (Bio-Rad). The intensity of each band was quantified with BioRad BImageLab 6.0 software.

### Active RAS pulldown (PD) assay

RAS pulldown assays were performed as previously described^76^. Briefly, cells were washed twice with ice-cold PBS, lysed with modified RIPA buffer, and cleared at 18,000 x g for 5 min at 4 °C. Total cell lysates (TCL) were normalized by the BCA assay. Non-denatured cell lysates were further subjected to PD, using GST fused to the RAS-binding domain (RBD) of the CRAF (known also as RAF1) kinase (GST-CRAF-RBD) as bait. PD and TCL samples were mixed with Laemmli buffer and denatured at 95 °C for 5 min. Immunoblotting analyses were performed using the antibodies indicated in figure and presented above.

### Quantitative real-time polymerase chain reaction (qRT-PCR)

Cells were seeded in a 6-well plate at a density of 250,000 cells/ml. Media was changed on the following day to osteogenic induction media +/- DOX. The media was changed at approximately 72 h intervals throughout the experiment and were analyzed on day 7. Total RNA was extracted using the RNA extraction kit (Qiagen) according to the manufacturer´s instructions. Reverse transcription was carried out using the High-Capacity Complementary DNA (cDNA) Reverse Transcription Kit (ThermoFisher Scientific). qRT-PCR was performed using the ViiA 7 Real-time PCR System (Life Technologies) with Platinum SYBR Green Mastermix (Invitrogen). Data were analyzed using the ΔΔCT method with Tbp and Hprt as a housekeeping gene. Primer sequences used were as follows: Opn (forward: GCTTGGCTTATGGACTGAGGTC; reverse: CCTTAGACTCACCGCTCTTCATG), Runx2 (forward: TGTTCTCTGATCGCCTCAGTG; reverse: CCTGGGATCTGTAATCTGACTCT), Sp7 (forward: CCCACCCTTCCCTCACTCAT; reverse: CCTTGTACCACGAGCCATAGG), Alp (forward: CCAGCAGGTTTCTCTCTTGG; reverse: CTGGGAGTCTCATCCTGAGC), Col1A1 (forward: GCTCCTCTTAGGGGCCACT; reverse: CCACGTCTCACCATTGGGG), Tbp (forward: GAAGCTGCGGTACAATTCCAG; reverse: CCCCTTGTACCCTTCACCAAT), Hrpt (forward: AGGGCATATCCAACAACAAACTT; reverse: GTTAAGCAGTACAGCCCCAAA).

### Statistical analysis

GraphPad Prism software (version 10) was used to calculate the statistical results of mean ± SD in the experiment. Two-way ANOVA followed by Tukey post-hoc test or Sidak´s multiple comparisons test for multiple comparisons were performed to analyze datasets. A p value less than 0.05 was considered to be statistically significant, *P < 0.05, **P < 0.01, ***P < 0.001.

## Electronic supplementary material

Below is the link to the electronic supplementary material.

Supplementary Material 1

## Data Availability

Data is provided within the manuscript or supplementary information files.
