# Publisher Correction: Impaired MC3T3-E1 osteoblast differentiation triggered by oncogenic HRAS is rescued by the farnesyltransferase inhibitor Tipifarnib

**DOI:** 10.1038/s41598-025-98727-0

**Published:** 2025-04-22

**Authors:** Yannik Andrasch, Moses Munene Ireri, Jonas Gander, Ann-Engelke Sabrina Timm, Saravanakkumar Chennappan, Miray Fidan, Melanie Engler, Ion Cristian Cirstea

**Affiliations:** 1 Institute of Comparative Molecular Endocrinology, Ulm University, Ulm, Germany; 2 Masonic Medical Research Institute, Utica, NY USA; 3 Institute of Applied Physiology, Ulm University, Ulm, Germany

Correction to: *Scientific Reports* 10.1038/s41598-025-91592-x, published online 26 February 2025

The original version of this Article contained an error in the Equal contribution statement.

“Moses Munene Ireri, Jonas Gander, Melanie Engler and Ion C. Cirstea contributed equally to this work.”

now reads:

- These authors contributed equally: Moses Munene Ireri and Jonas Gander
- These authors jointly supervised this work: Melanie Engler and Ion Cristian Cirstea

The original Article has been corrected.

