# Supplementary material for: Impaired MC3T3-E1 osteoblast differentiation triggered by oncogenic HRAS is rescued by the farnesyltransferase inhibitor Tipifarnib
Source: Sci Rep. 2025 Feb 26;15:6832. doi: 10.1038/s41598-025-91592-x (PMC11861272; doi:10.1038/s41598-025-91592-x)

Impaired MC3T3-E1 osteoblast differentiation triggered by oncogenic HRAS is rescued by the farnesyltransferase inhibitor Tipifarnib.

Yannik Andrasch[1], Moses Munene Ireri[1,3,*], Jonas Gander[3,*], Ann-Engelke Sabrina Timm[1], Saravanakkumar Chennappan[2], Miray Fidan[1], Melanie Engler[# 1,3], Ion C. Cirstea[# 1,3]

[1], Institute of Comparative Molecular Endocrinology, Ulm University, Ulm, Germany

[2], Masonic Medical Research Institute, Utica, NY, United States.

[3], Institute of Applied Physiology, Ulm University, Ulm, Germany.

[*] These authors contributed equally to this work.

[#] These authors contributed equally to this work.

Correspondence: Ion Cristian Cirstea, ion.cirstea@uni-ulm.de

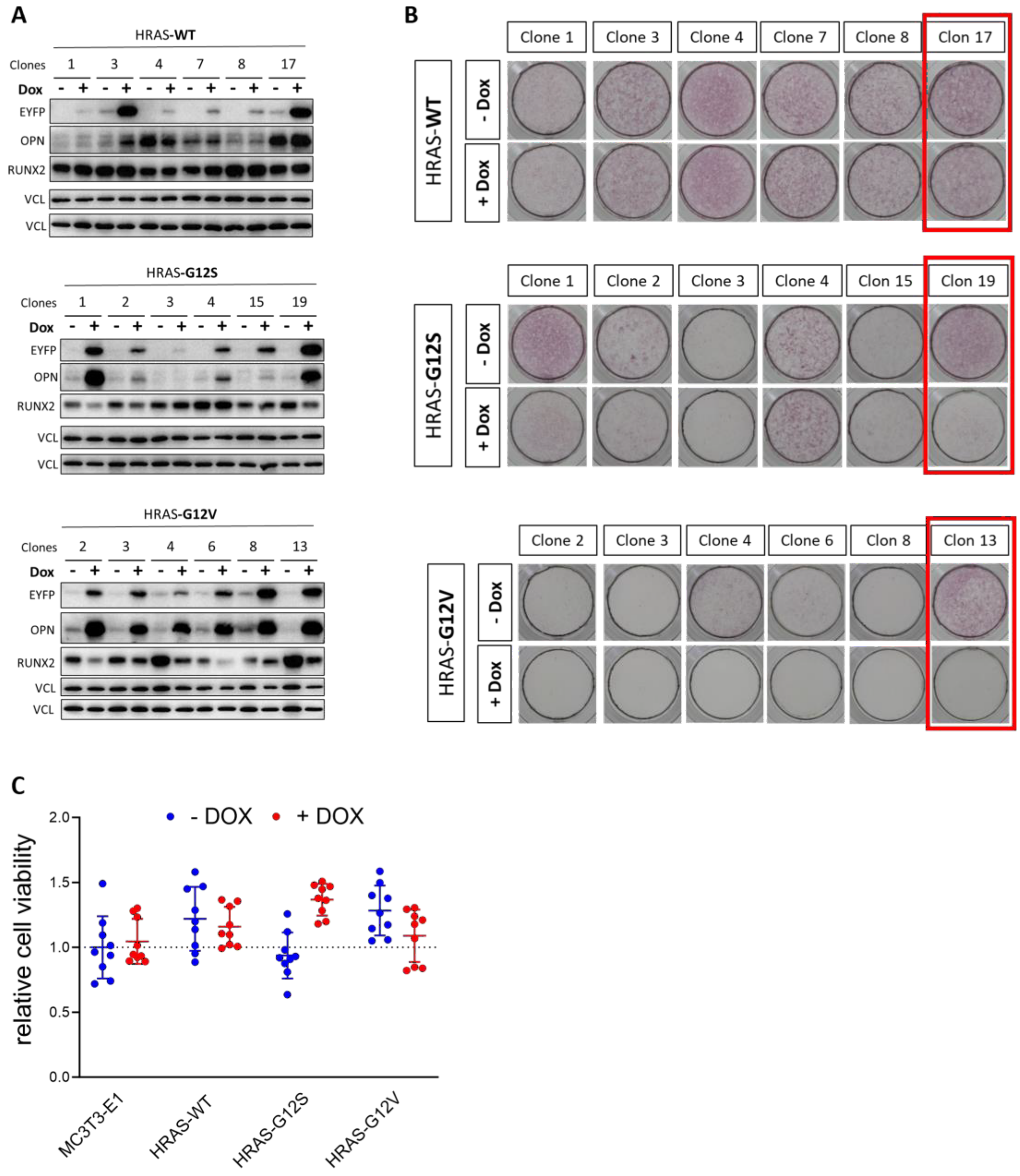

Supplementary Figure 1. (A) Screening of recombinant HRAS expression in HRAS WT, HRAS G12S and HRAS G12V MC3T3-E1 clones generated by lentiviral transduction. Recombinant HRAS expression was analyzed by immunoblotting with GFP specific antibody, while vinculin (VCL) serves as loading control. Original blots are presented in Supplementary Figure 11. **(B)** Quantitative assessment of osteoblast differentiation in HRAS WT, HRAS G12S and HRAS G12V MC3T3-E1 clones by Alp activity was measured using Alp assay kit, 7 days after osteogenic induction. Clones that were selected for the study are highlighted in red. **(C)** Cell viability, of selected clones in response to DOX and osteogenic induction, was measured after 7 days using PrestoBlue assay.

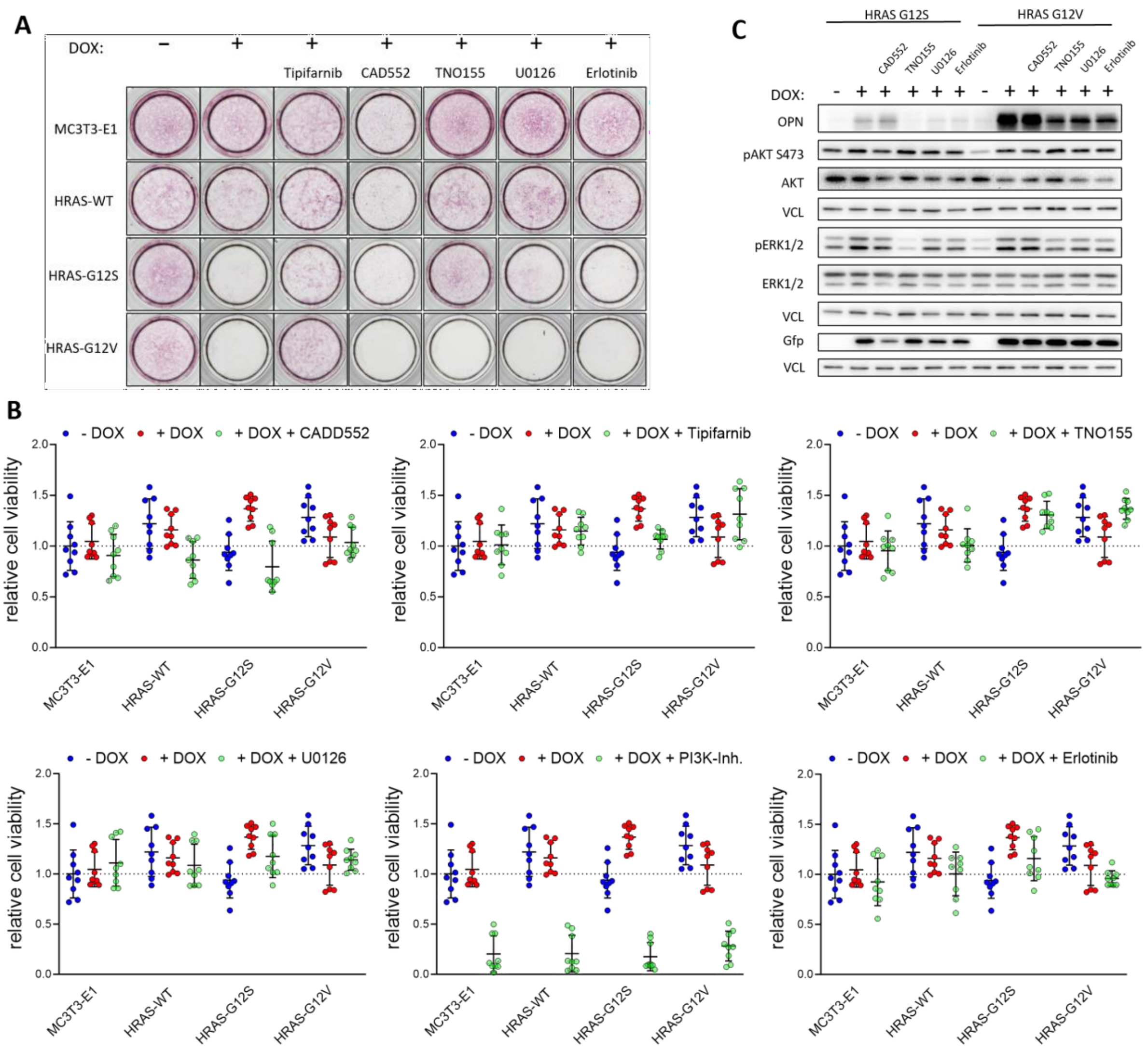

Supplementary Figure 2. (A) Differentiation of parental MC3T3-E1 and MC3T3-E1 cells expressing the recombinant HRAS constructs in response to DOX and inhibitors treatment: FTase inhibitor (Tipifarnib; 0,5 µM), RUNX2 inhibitor (CADD522; 50 µM), SHP2 inhibitor (TNO155; 1 µM), MEK1/2 inhibitor (U0126; 10 µM) or EGFR inhibitor (erlotinib; 5 µM). ALP activity was analyzed 7 days post osteogenic induction. Qualitative assessment was performed by imaging 24-well plates with Canon Sigma DG macro camera (n=3). **(B)** Cell viability as measured by Presto Blue. Cells were kept in osteogenic induction media for 7 days in the presence or absence of DOX and indicated inhibitors, with daily media change. **(C)** Analyses of Erk1/2 and Akt activation and the expression of Opn by immunoblotting of samples from MC3T3-E1 subclones harboring HRAS G12S and HRAS G12V in the presence or absence of DOX and the RUNX2 inhibitor (CADD522 50 µM), SHP2 inhibitor (TNO155 1 µM), MEK1/2 inhibitor (U0126 10 µM) or EGFR inhibitor (erlotinib 5 µM), with specific antibodies for activating phosphorylations of Erk1/2, Akt (serine 473 and threonine 308) and Opn. Akt and Erk1/2 blots were used as control for their phospho-forms, whereas Vcl serves as loading control and GFP as control for expression of recombinant protein.

Supplementary figure 3. Original blots for Figure 1b.

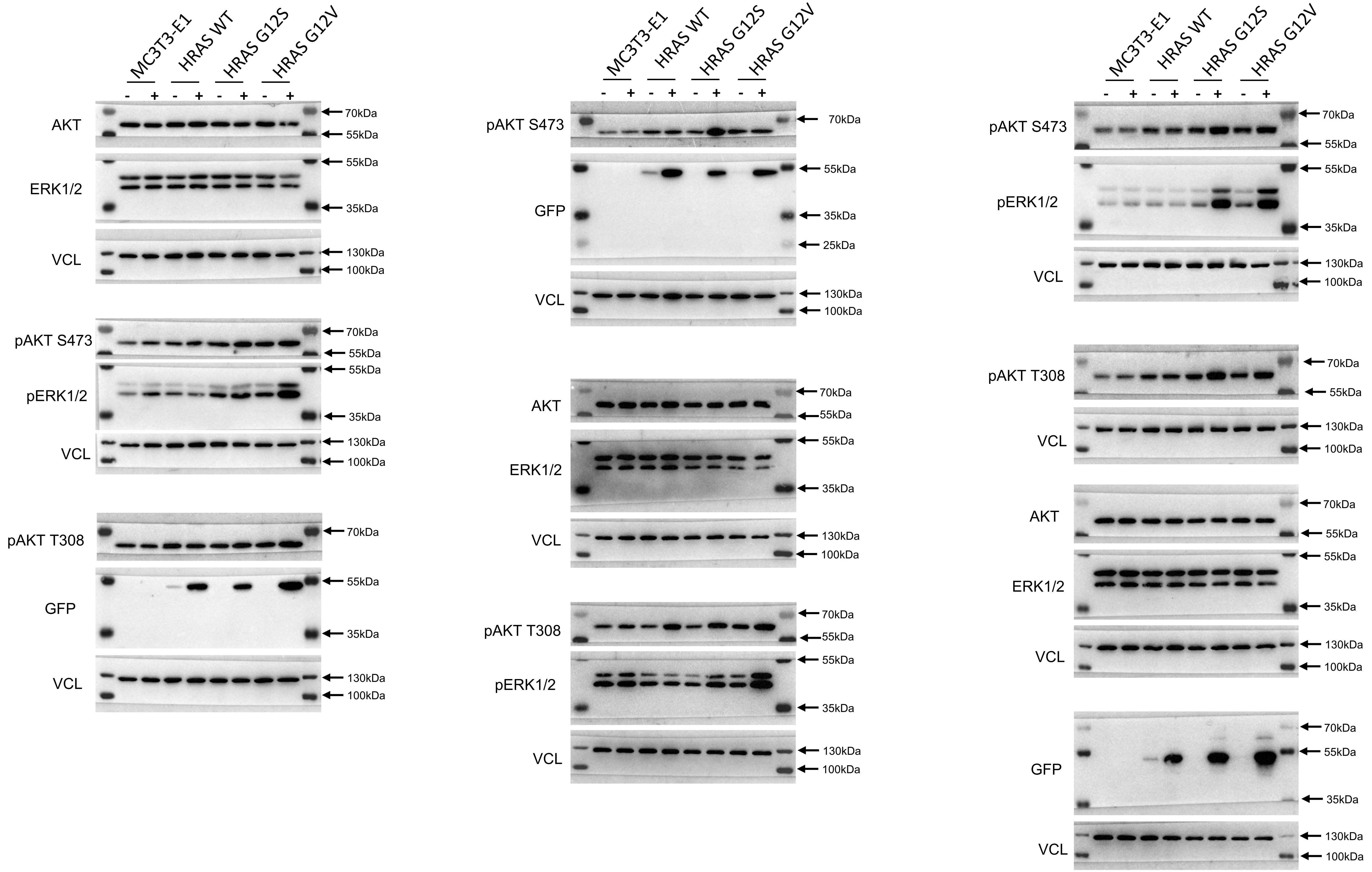

Supplementary figure 4. Original blots for Figure 1f.

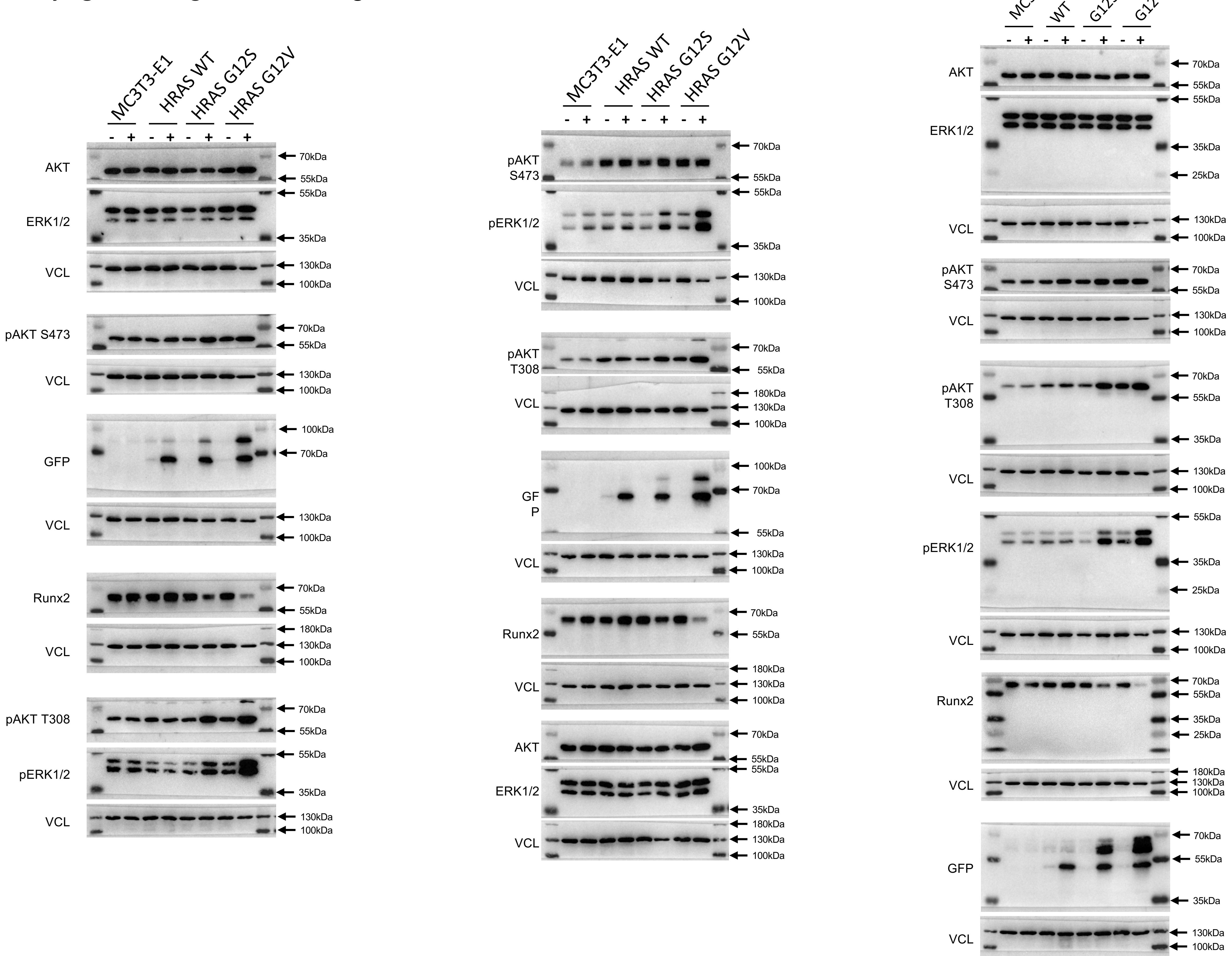

Supplementary figure 5. Original blots for Figures 2d and 2e.

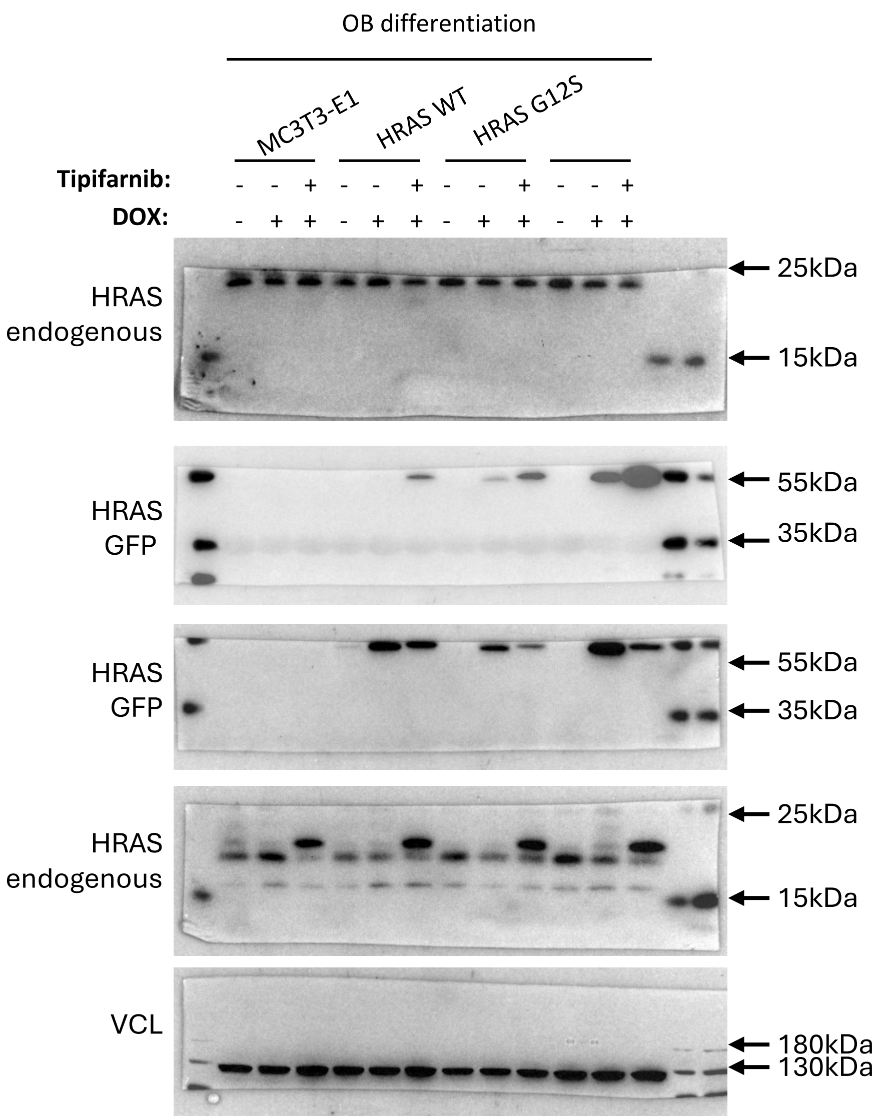

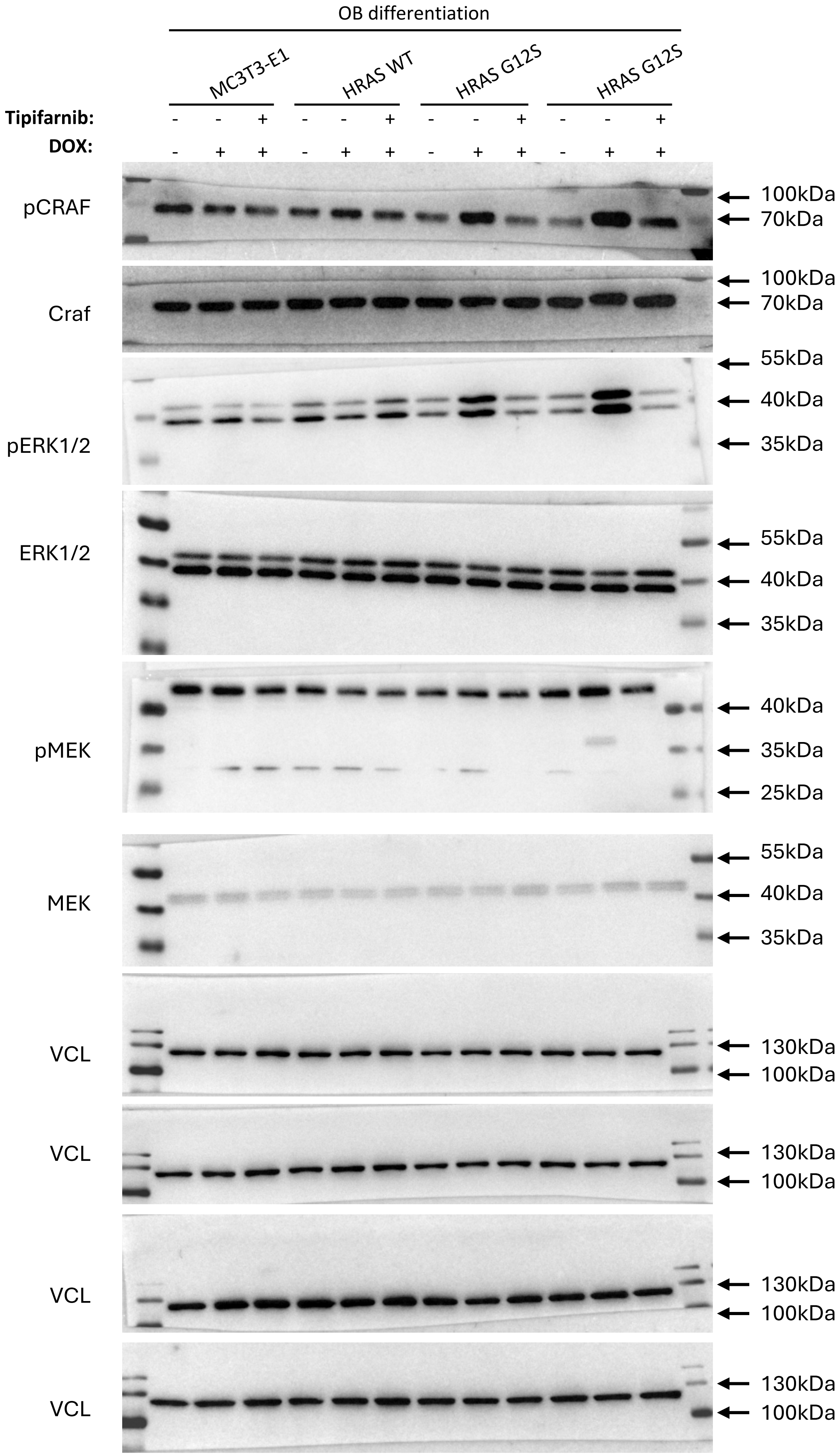

Supplementary figure 6. Original blots for Figure 3a.

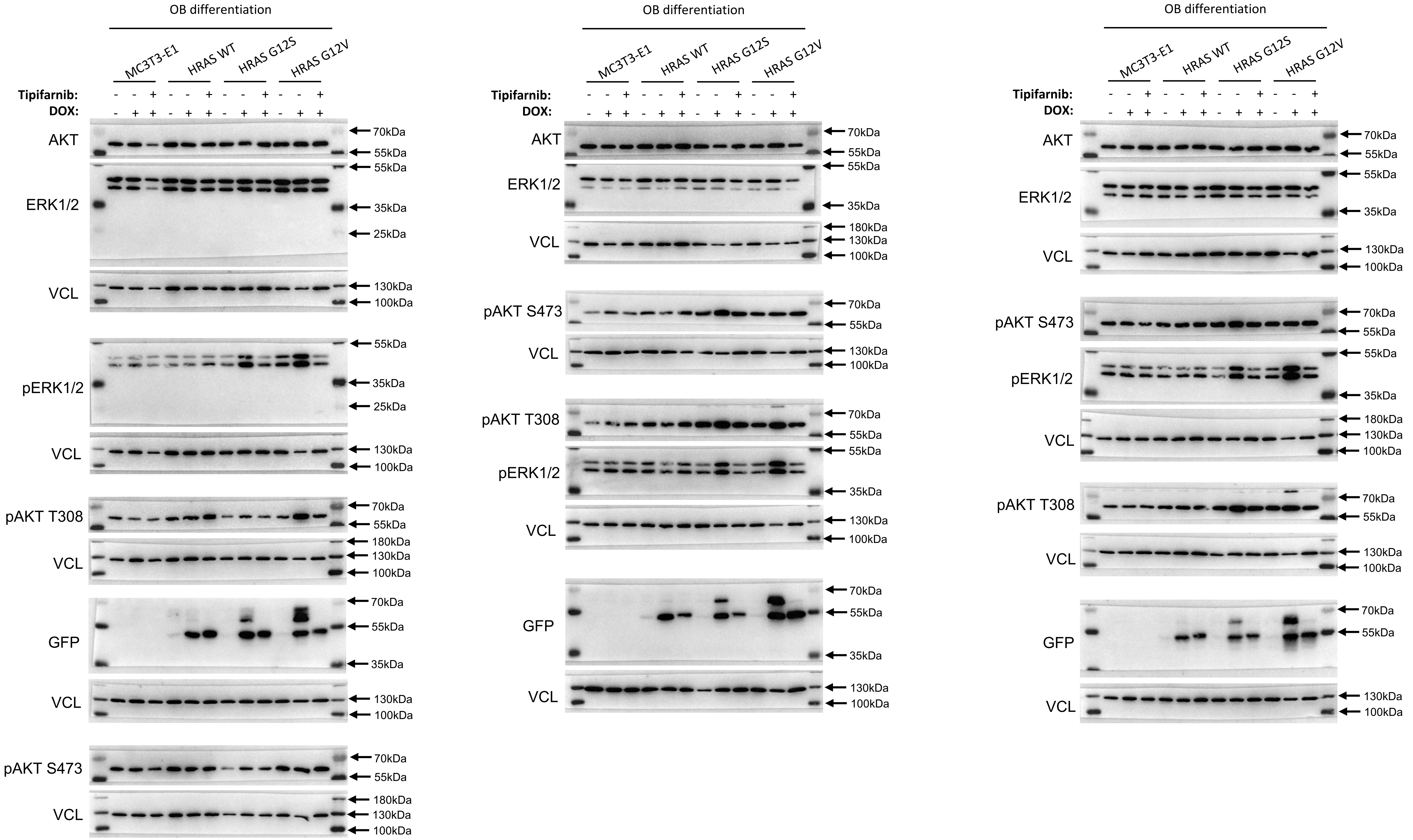

Supplementary figure 7. Original blots for Figure 3c.

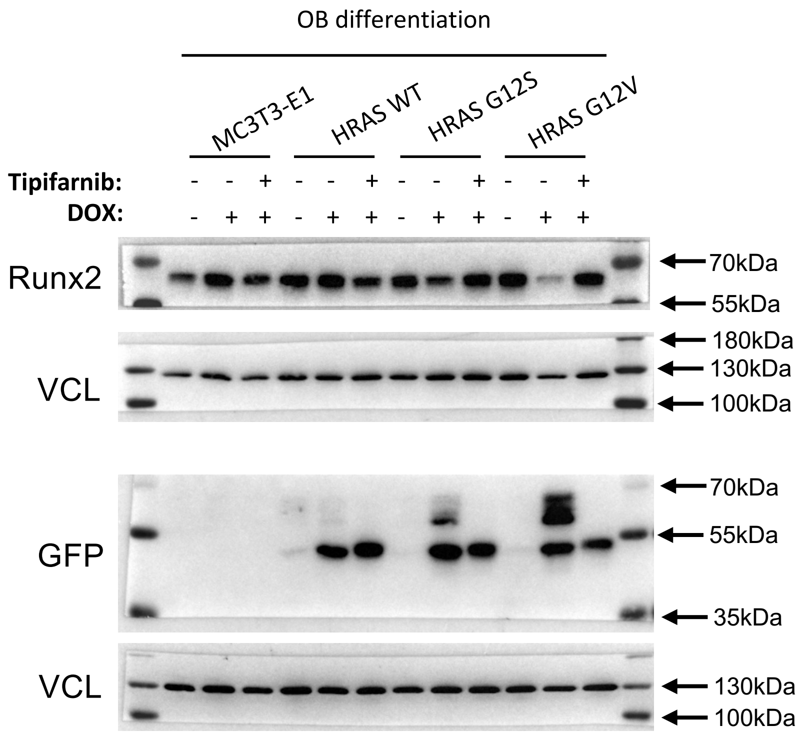

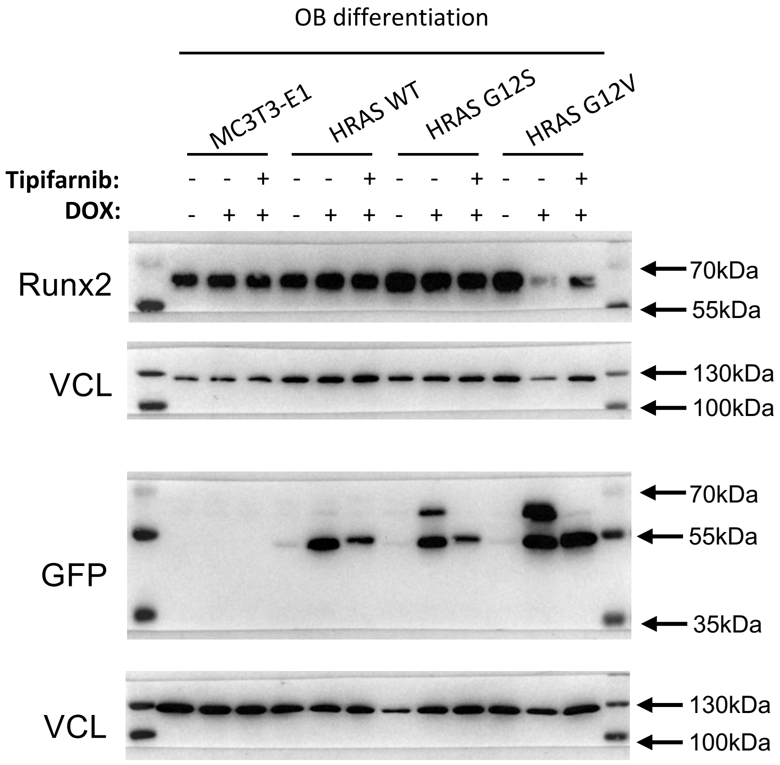

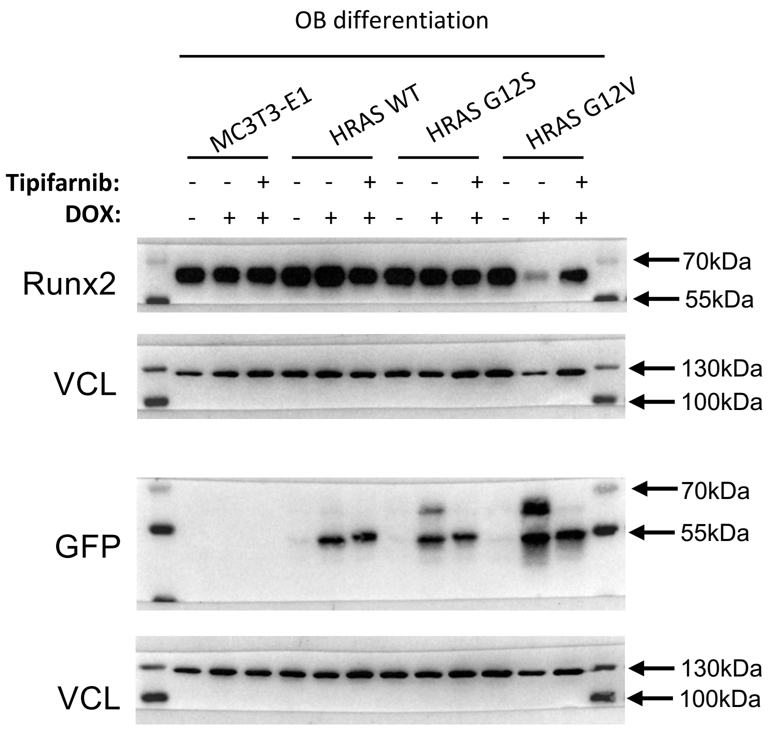

Supplementary figure 8. Original blots for Figure 4a.

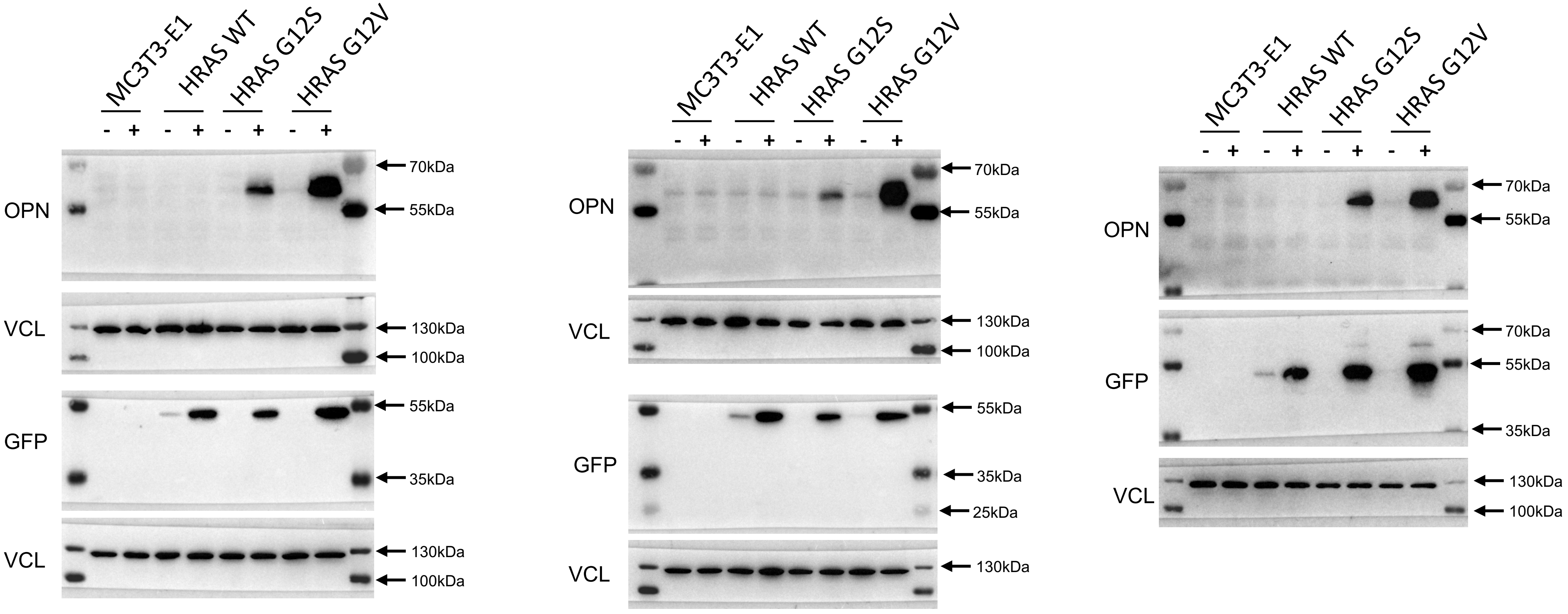

Supplementary figure 9. Original blots for Figure 4b.

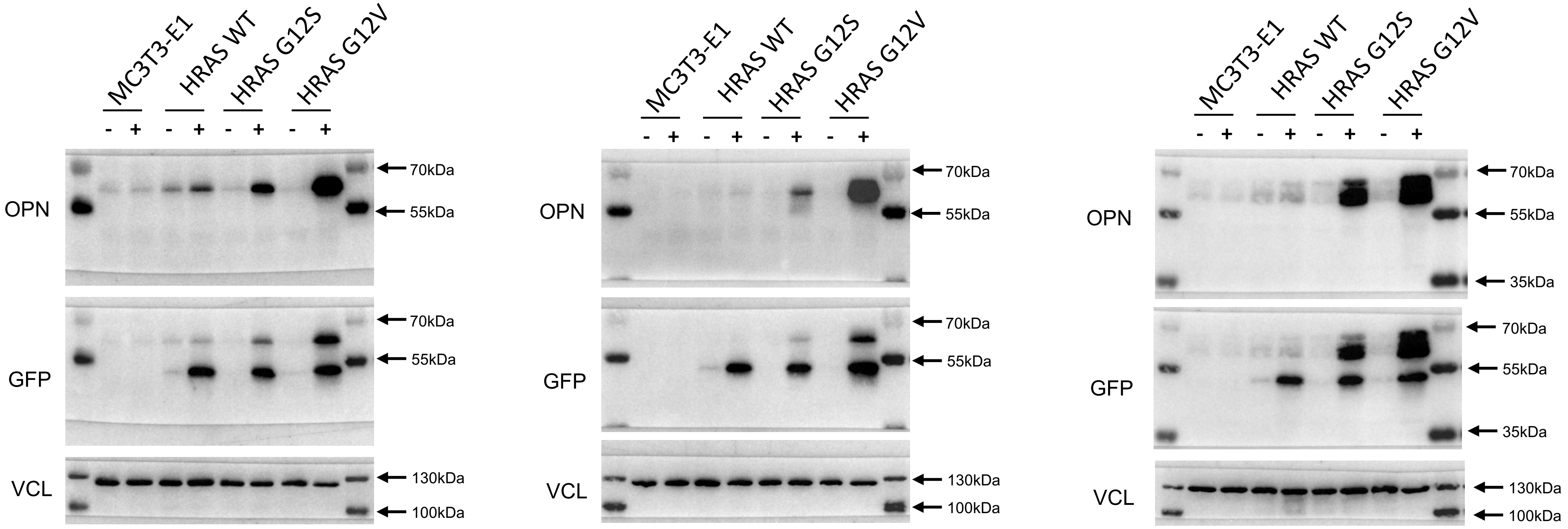

Supplementary figure 10. Original blots for Figure 4g.

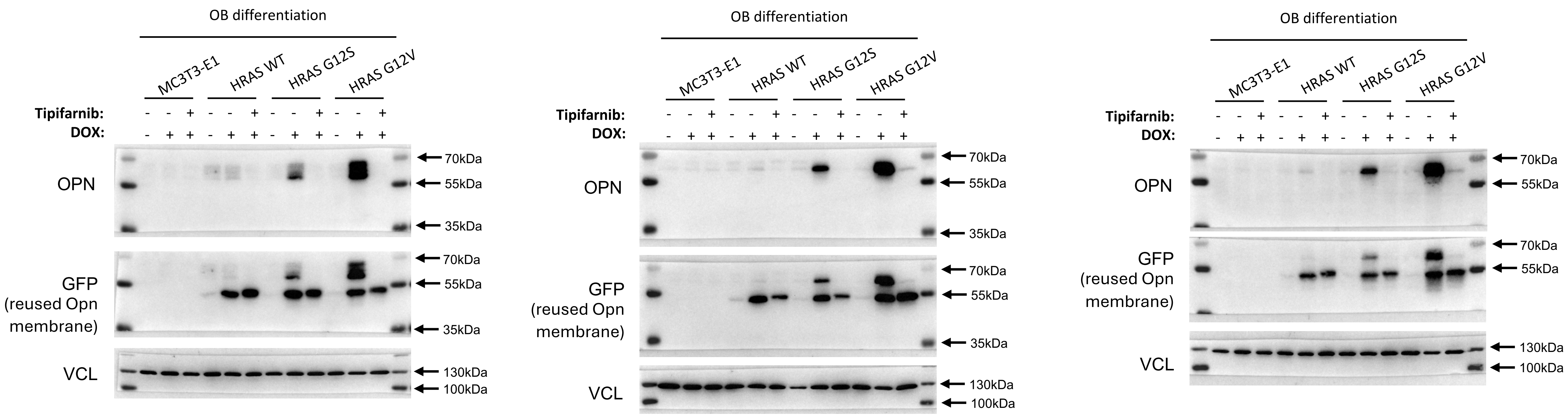

Supplementary figure 11. Original blots for Supplementary Fig. 1a

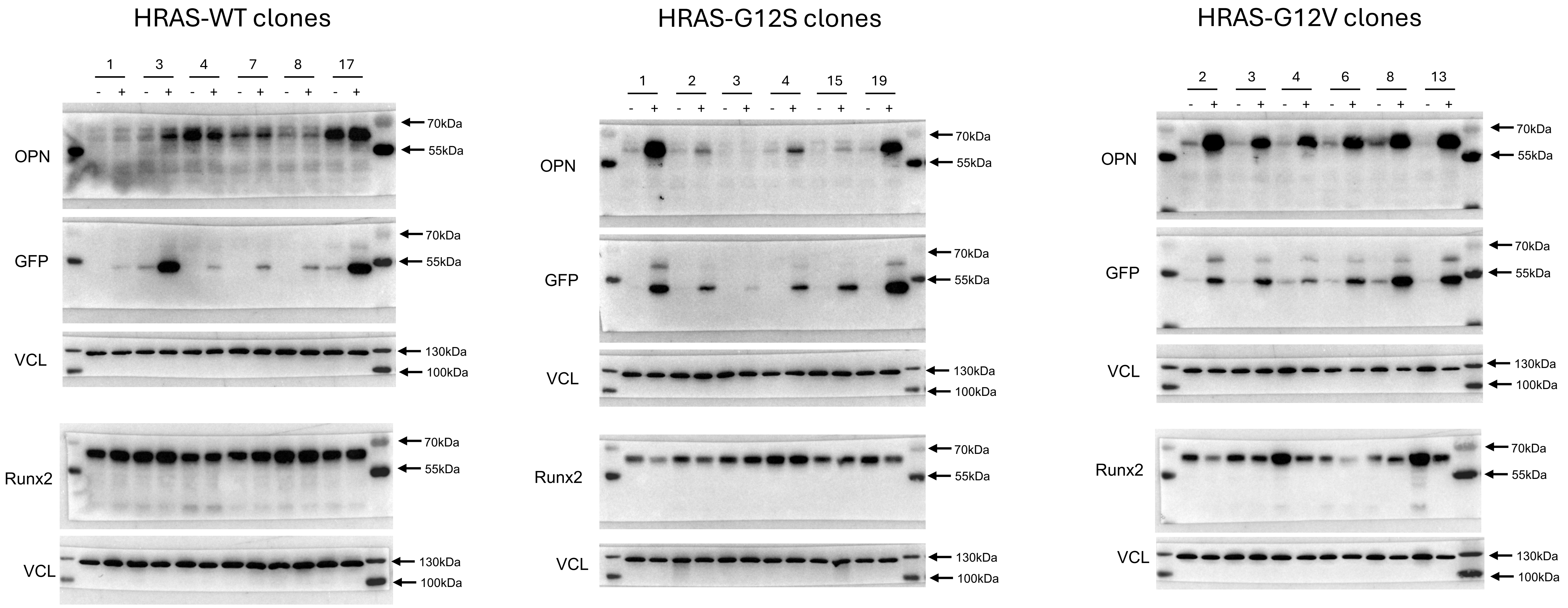

Supplement: Supplementary file 1 — Supplementary Material 1 [file 41598_2025_91592_MOESM1_ESM.pdf]
